# Cerebral Microbleeds Were Related With Poor Cognitive Performances on the Dual Task Condition in Older Adults

**DOI:** 10.3389/fnagi.2021.807753

**Published:** 2022-01-10

**Authors:** Xuanting Li, Shuna Yang, Wei Qin, Lei Yang, Yue Li, Yutong Hou, Qixin Huang, Wenli Hu

**Affiliations:** Department of Neurology, Beijing Chao-Yang Hospital, Capital Medical University, Beijing, China

**Keywords:** cerebral microbleed, dual task, cognitive-motor interference, cognition, motor

## Abstract

**Background:** The dual task (DT) was commonly used to assess the risk of falls in older adults and patients with neurological disorders. However, the performance on DT conditions has not been well investigated in patients with cerebral microbleed (CMB). This study is aimed to compare the performance in DT tests between older adults with and without CMB, and to explore the association between CMB and cognitive performances of DT.

**Methods:** This is a cross-sectional study. A total of 211 old adults participated, involving 68 CMB patients. The task protocol involved two global cognition tests, two single cognitive tests (serial 7 subtraction and semantic fluency), two single motor tasks [8-m walking and timed up and go test (TUG)], and three DT tests [walking and serial subtraction (WSS), walking and semantic fluency (WSF), and TUG and serial subtraction (TUGSS)]. The time taken to complete each task and the number of correct responses were recorded. For each DT condition, the correct response rate (CRR) and the dual-task effect (DTE) for the correct number were calculated.

**Results:** Compared with subjects without CMB, CMB patients had worse cognitive performances on DT condition in CRR of WSS (*p* = 0.003), WSF (*p* = 0.030) and TUGSS (*p* = 0.006), and DTE of WSS (*p* = 0.017). Binary logistic regression analysis showed that the presence of CMB was an independent risk factor for the impairment group for CRR of TUGSS (*OR*, 2.54; 95% CI, 1.11–5.82; *p* = 0.027) with the adjustment for confounders, rather than CRR of WSS and WSF, or DTE of WSS. Multiple linear regression analysis showed that CRR of TUGSS decreased with the increase of CMB number grades (β, −0.144; 95% CI, −0.027, −0.002; *p* = 0.028).

**Conclusion:** The present study indicated that CMBs were closely associated with poor cognitive performances on DT in the elderly. Strongest effect size was seen for CRR of TUGSS, where performance deficits increased in proportion to the degree of CMB burden.

## Introduction

Dual task (DT) refers to a paradigm that an individual performs two attention-demanding tasks with different goals simultaneously. Taking two tasks concurrently has negative effects on the performance of both tasks (Nonnekes et al., 2020). The difference between the performance on each single task and DT provides an index of DT ability. In people's daily life, performing various activities requires the coordination of complex cognitive and motor functions. And the one-dimensional cognitive or motor test may not be accurate enough to assess people's ability of daily living. Thus, the DT may be an important method for the complemental assessment of physical function.

Previous studies have reported that the performance in DT tests of the elderly is significantly worse than that of the young (Lindenberger et al., 2000; Pothier et al., 2015; Papegaaij et al., 2017). White matter hyperintensity (WMH), a kind of aging-related small vessel lesions that is common in older adults, has been found a close relationship with poor gait performances on the DT condition (Ghanavati et al., 2018; Hairu et al., 2021). It's suggested that cerebral small vessel disease (CSVD) may be an underlying cause of poor DT performances in the elderly. Cerebral microbleed (CMB) is one of the crucial markers of CSVD, and its pathological mechanism is closely related to hypertension and cerebral amyloid angiopathy (Shuaib et al., 2019). Many studies have indicated that CMB is associated with the cognitive impairment or gait dysfunction (Akoudad et al., 2016; Chiu et al., 2018; Nyúl-Tóth et al., 2020; Li et al., 2021a; Sullivan et al., 2021). However, there are no studies specifically focusing on the effect of CMB lesions on DT performances.

The purpose of the current study is to investigate how performances of a cognitive-motor DT protocol differ between older adults with and without CMB, and to prove the association between CMB and cognitive performances under the DT condition. We hypothesize that the DT performance of old adults with CMB is worse than that of those without CMB, and that the presence and the severity of CMB are closely related to the cognitive performance decline in DT.

## Methods

### Subjects

This study was designed as a cross-sectional study. Participants for physical examinations were recruited in the Neurology Department of Beijing Chao-yang Hospital, Capital Medical University from January 2021 to September 2021. Written informed consent was obtained from eligible participants. The study was performed in compliance with the Declaration of Helsinki and approved by the Ethics Committee of Beijing Chao-yang Hospital, Capital Medical University (2021-Sci-56).

Inclusion criteria were age ≥60 years, available clinical data and brain magnetic resonance imaging (MRI), and agreement to participate in this study. The exclusion criteria included: (1) acute cerebrovascular diseases such as acute cerebral infarction, cerebral hemorrhage, venous sinus thrombosis and so on; (2) history of the massive relevant cerebral infarction (recent infarct > 2.0 cm in diameter and the correspondent lacuna > 1.5 cm in diameter) and cerebral hemorrhage with neurological sequelae; (3) the neurodegenerative disease (Parkinson's disease, Alzheimer's disease, multiple system atrophy, etc.), history of nervous system infection, inflammatory demyelinating disease, brain trauma, poisoning, radioactive encephalopathy and metabolic encephalopathy; (4) severe neuropsychological diseases and mental illnesses affecting the results of cognitive function assessments; (5) patients with cardiac insufficiency, hepatic failure, kidney failure or other medical conditions that were too weak prevented the patients from performing a proper assessment; (6) orthopedic conditions or pain affecting natural motor function; (7) taking cognitive-affecting drugs within 24 h; (8) severe visual or hearing impairment; (9) incomplete data of clinical records, motor function assessments and neuropsychological tests, or brain MRI with poor quality.

Baseline information including age, gender, years of education, height, weight, smoking and drinking status, medication use and medical history of hypertension, diabetes, hyperlipidemia, stroke, transient ischemic attack, or coronary artery disease was collected from all participants according to medical records and questionnaires.

### Neuroimaging Assessments

MRI was performed on a 3.0-T MRI scanner (Prisma; Siemens AG, Erlangen, Germany). The parameters of the MRI examination were as follows: T1-weighted imaging (repetition time = 2000.0 ms, echo time = 9.2 ms, slice thickness = 5 mm, and field of view = 220 × 220 mm^2^), T2-weighted imaging (repetition time = 4,500.0 ms, echo time = 84.0 ms, slice thickness = 5.0 mm, and field of view = 220 × 220 mm^2^), T2-weighted fluid-attenuated inversion recovery sequence (repetition time = 8,000.0 ms, echo time = 86.0 ms, slice thickness = 5.0 mm, and field of view = 199 × 220 mm^2^), diffusion-weighted imaging (repetition time = 3,300.0 ms, echo time = 91.0 ms, slice thickness = 5.0 mm, field of view = 230 × 230 mm^2^, and b = 0 and 1,000 s/mm^2^), and susceptibility-weighted imaging (repetition time = 27.0 ms, echo time = 20.0 ms, slice thickness = 3.2 mm, and field of view = 172 × 230 mm^2^).

Main neuroimaging markers of CSVD were defined according to the STandards for ReportIng Vascular changes on nEuroimaging published previously (Wardlaw et al., 2013). The location and number of CMB were collected based on the Microbleed Anatomical Rating Scale (Gregoire et al., 2009). And the severity of CMB was classified into four grades by the number of lesions (0 CMB, 1 CMB, 2 CMBs and ≥3 CMBs). The paraventricular hyperintensity (PVH) and deep white matter hyperintensity (DWMH) were graded according to the Fazekas scale ranging from 0 to 3, respectively (Fazekas et al., 1987). The burden of perivascular space (PVS) in centrum semiovale and basal ganglia (BG) was evaluated separately (grade 0 to 4) (Maclullich et al., 2004; Li et al., 2021b). The visual rating scale for posterior atrophy was used to assess the severity of brain atrophy ranging from 0 to 3 (Koedam et al., 2011). Moreover, we noted the total number and distribution of lacuna lesions.

Neuroimaging markers of CSVD were identified and labeled by consensus of two experienced neurologists blinded to clinical data. Disagreement was resolved by discussing with other coauthors.

### Procedures

#### Cognitive Tests

Global cognition was assessed using the Mini-Mental State Examination (MMSE) and Montreal Cognitive Assessment (MoCA). Besides, the serial 7 subtraction task and semantic fluency task (animals) in MoCA were selected as two kinds of single cognitive tasks. For the serial 7 subtraction task, subjects were asked to calculate at least 60 s and at least five times. If the subject cannot perform the subtraction, the time for this item was limited to 60 s. For the semantic fluency task, subjects were asked to generate as many nouns as possible within 60 s. Besides, we recorded the subjects' responses and noted the number of correct answers. Cognitive assessments and DT tests were conducted within 5 days after the MRI scan.

#### Motor and Dual Tasks

Subjects performed all the following five tasks walking over a tiled floor. On the DT condition, subjects walked with the instruction to “perform both tasks as well as possible without giving priority to either motor or cognitive task”. Subjects' responses were recorded, and we also noted the number of correct responses.

1. Eight-meter walking task: Participants were asked to walk on level ground along an 8-m pathway at their usual and comfortable pace.
2. Walking and serial subtraction task (WSS): Participants walked along the 8-m walkway while repeatedly subtracting 7 from a random number concurrently.
3. Walking and semantic fluency task (WSF): Participants were instructed to walked along the 8-m walkway while doing semantic fluency task (vegetables) concurrently.
4. Timed up and go test (TUG): Participants were asked to stand up from a chair, walk 3 m, turn 180°, then walk back, and sit down at their usual and comfortable speed.
5. TUG and serial subtraction task (TUGSS): Participants performed the TUG test and serial 7 subtraction task from a random number simultaneously.

To avoid the learning effects, the rater assigned different word categories (animals or vegetables) or random numbers (between 80 and 100) when assessing the single cognitive test and the DT test. And our pilot study found that different sets of numbers or word categories produced comparable difficulty among participants.

Subjects' motor data were captured using the Intelligent Device for Energy Expenditure and Activity (IDEEA®, MiniSun LLC) system. The main recorder of IDEEA system was secured on the left waistband. One sub-recorder was taped above each lateral malleolus. Five sensors were placed on the sternum and bilaterally on the plantar aspect of foot and midline of the anterior aspect of thigh. The IDEEA system automatically recognized the movement of body and the change of posture, and recorded various parameters of tests including the start and end time, duration, gait, etc. The data was downloaded from the recorder to the computer after performing tests on each subject. Two gait parameters on each DT condition, the walking speed (m/s) and stride length (m), were included in this analysis.

#### Dual Task Performance Assessments

The correct response rate (CRR) was calculated using the duration and the number of correct responses in each DT test for measuring the cognitive performance under the DT condition. The CRR was computed as (Yang et al., 2016):


$$\begin{array}{l} CRR=\;\frac{number\;of\;correct\;reponses}{time} \end{array}$$

We used the dual-task effect (DTE) to assess the influence of the added motor task on the cognitive performance. The DTE was computed as (Kelly et al., 2010):


$$\begin{array}{l} DTE,\;\%=\;\frac{dual\;task\;-\;single\;task}{single\;task}\;\times 100\% \end{array}$$

In this study, we calculated the DTE for the number of correct responses. The time limit given to count the answers in each single cognitive task was matched to the duration of the corresponding DT test. For example, if it took the subject 30 s to perform the DT of walking and subtraction, then we noted the number of correct responses in the first 30 s in the single task of subtraction in MoCA according to the recording. The negative value of the DTE indicates worse cognitive performance on the DT condition compared with the single task condition (“cognitive costs”), while the positive value indicates the improvement of performances (“cognitive benefits”) (Al-Yahya et al., 2011; Pumpho et al., 2020).

The increased time of WSS, WSF and TUGSS was calculated as the duration of WSS minus the duration of 8-m walking, the duration of WSF minus the duration of 8-m walking, and the duration of TUGSS minus the duration of TUG task, respectively.

The increased numbers of WSS, WSF and TUGSS were calculated as the correct number of WSS minus the correct number of single subtraction task, the correct number of WSF (vegetables) minus the correct number of semantic fluency (animals), and the correct number of TUGSS minus the correct number of single subtraction task, respectively. The time to count responses in each single cognitive task was matched to the duration of the corresponding DT test.

Impaired cognitive performance on DT was defined as the lowest quartile of CRR or DTE. Those participants with impaired cognitive performance were considered as the impairment group, the others were assigned to the control group.

### Statistical Analyses

Data were presented as n (%) for categorical variables, mean (standard deviation) for normally distributed variables, or median (quartiles) for continuous data with non-normal distribution. The severity grade of WMH, PVS and PA was represented by the median (range). Differences among groups were determined using χ^2^, Mann-Whitney U or Kruskal-Wallis H test where appropriate.

The univariate analysis was used to identify the statistical differences of parameters in the cognitive performance between CMB patients and non-CMB subjects. Next, subjects were divided into the impairment group and the control group according to each parameter with statistical difference, separately. Then, age, sex, years of education and variables with significant differences between the impairment group and control group were adjusted in the following regression analysis as confounding factors.

Binary logistic regression analysis was performed to determine whether the presence of CMB was an independent risk factor of the DT performance. Multiple linear regression analysis was used to explore the trend of the DT performance with the increase of CMB number grades. All analyses were performed with Statistical Product and Service Solutions 22.0, and the statistical significance was considered at *p* < 0.05.

## Results

Sample characteristics are depicted in Table 1. A total of 211 elderly subjects were recruited in this study, including 116 males and 95 females with the median age of 70 years old. There were 68 patients with CMB and 143 subjects without CMB. As for the location of CMB, 24 patients had strictly lobar CMB, 16 patients had strictly deep CMB, two patients had strictly infratentorial CMB (cerebellum or brain stem, or both), 12 patients had mixed CMB (lobar, deep and infratentorial), 6 patients had mixed CMB (lobar and deep), 5 patents had mixed CMB (lobar and infratentorial), and 3 patients had mixed CMB (deep and infratentorial). There were 83 subjects with lacuna, including 32 patients with one lesion and 51 with multiple lesions. Six patients had lobar lacuna located in the cerebral lobe, 57 had lacuna in the basal ganglia, 39 had paraventricular lesion, and 20 had infratentorial lesion.

**Table 1:** Characteristics of the study population.

	**All participants (*n* = 211)**
Age^a^, year	70.00 (64.00, 76.00)
Men, *n* (%)	116 (54.98)
Years of education^a^, year	9 (9, 12)
Height^a^, m	1.65 (1.59, 1.70)
Weight^a^, kg	69.00 (60.00, 75.00)
MMSE^a^	28 (26, 29)
MoCA^a^	24 (22, 26)
Smoking, *n* (%)	65 (30.81)
Drinking, *n* (%)	36 (17.06)
Hypertension, *n* (%)	147 (69.67)
Diabetes mellitus, *n* (%)	63 (29.86)
CAD, *n* (%)	50 (23.70)
History of stroke/TIA, *n* (%)	46 (21.80)
Hyperlipemia, *n* (%)	76 (36.02)
Blood pressure-lowering medication, *n* (%)	129 (61.14)
Antiplatelet/anticoagulant drug, *n* (%)	66 (31.28)
Antidiabetic drug, *n* (%)	60 (28.44)
Lipid-lowering drug, *n* (%)	68 (32.23)
CSVD MRI markers	
Presence of lacune, *n* (%)	83 (39.34)
Presence of CMB, *n* (%)	68 (32.23)
PVH^b^	1 (1–3)
DWMH^b^	1 (0–3)
BG-PVS^b^	1 (1–4)
CSO-PVS^b^	2 (1–4)
PA ^b^	1 (0–2)

*MMSE, mini-mental state examination; MoCA, Montreal cognitive assessment; CAD, coronary artery disease; TIA, transient ischemic attack; CSVD, cerebral small vessel disease; MRI, magnetic resonance imaging; CMB, cerebral microbleed; PVH, periventricular hyperintensity; DWMH, deep white matter hyperintensity; BG, basal ganglia; CSO, centrum semiovale; PVS, perivascular space; PA, posterior atrophy*.

a *Median (quartiles)*.

b *Median (range)*.

Compared with non-CMB subjects, CMB patients had lower scores of MMSE (*p* = 0.012) and MoCA (*p* = 0.001), and worse cognitive performances on DT in CRR of WSS (*p* = 0.003), WSF (*p* = 0.030) and TUGSS (*p* = 0.006), and DTE of WSS (*p* = 0.017). There were no significant differences in the increased correct number or time of WSS, WSF and TUGSS, or DTE of WSF and TUGSS between CMB patients and non-CMB subjects (all *p* > 0.050) (Table 2). In CMB patients, numbers of correct answers of WSS and TUGSS were significantly lower than those of time-matched single subtraction tasks [1 (0, 2) vs. 2(1, 4), *p* < 0.001; 1 (0, 3) vs. 3 (1, 4), *p* < 0.001], while no significant difference was observed in the semantic fluency [7 (5, 8) vs. 7 (6, 8), *p* = 0.713]. After comparing gait parameters on each DT condition between two groups, CMB patients had slower stride speeds and shorter stride lengths, while the differences were statistically significant only in the stride length in TUGSS (*p* = 0.045) (Table 2).

**Table 2:** Comparison of cognitive performances and gait parameters between participants with and without CMB.

	**CMB (*n* = 68)**	**No CMB (*n* = 143)**	***p***
MMSE^a^	27 (25, 29)	28 (27, 29)	0.012^*^
MoCA^a^	23 (21, 25)	25 (23, 26)	0.001^*^
Increased number of WSS^a^	−1.00 (−2.00, 0.00)	−1.00 (−2.00, 0.00)	0.161
Increased number of WSF^a^	0.00 (−2.00, 1.00)	−1.00 (−2.00, 1.00)	0.530
Increased number of TUGSS^a^	−1.00 (−2.75, 0.00)	−1.00 (−2.00, 0.00)	0.274
Increased time of WSS^a^, s	4.20 (2.50, 7.38)	3.47 (1.80, 7.10)	0.328
Increased time of WSF^a^, s	2.43 (1.24, 4.96)	2.20 (1.03, 4.28)	0.317
Increased time of TUGSS^a^, s	6.10 (2.68, 8.75)	4.27 (1.80, 7.50)	0.053
CRR of WSS^a^	0.06 (0.00, 0.16)	0.12 (0.04, 0.23)	0.003^*^
CRR of WSF^b^	0.49 (0.21)	0.56 (0.21)	0.030^*^
CRR of TUGSS^b^	0.08 (0.09)	0.12 (0.10)	0.006^*^
DTE of WSS^a^, %	−58.33 (−100.00, 0.00)	−33.33 (−75.00, 0.00)	0.017^*^
DTE of WSF^a^, %	0.00 (−29.64, 14.29)	−10.00 (−28.57, 20.00)	0.852
DTE of TUGSS^a^, %	−53.57 (−100.00, 0.00)	−33.33 (−80.00, 0.00)	0.052
Speed in WSS^a^, m/s	0.56 (0.38, 0.66)	0.58 (0.44, 0.77)	0.125
Stride length in WSS^a^, m	0.78 (0.65, 0.90)	0.83 (0.67, 0.97)	0.054
Speed in WSF^a^, m/s	0.60 (0.47, 0.76)	0.67 (0.49, 0.81)	0.189
Stride length in WSF ^b^, m	0.81 (0.20)	0.86 (0.18)	0.073
Speed in TUGSS^a^, m/s	0.44 (0.33, 0.53)	0.48 (0.37, 0.56)	0.051
Stride length in TUGSS^a^, m	0.63 (0.53, 0.79)	0.70 (0.57, 0.80)	0.045^*^

*CMB, cerebral microbleed; MMSE, mini-mental state examination; MoCA, Montreal Cognitive Assessment; WSS, walking and serial subtraction; WSF, walking and semantic fluency; TUGSS, timed up and go and serial subtraction; CRR, correct response rate; DTE, dual-task effect*.

a *Median (quartiles)*.

b *Mean (standard deviation)*.

* *P < 0.05*.

Subjects were divided into the impairment group (the lowest quartile of CRR or DTE) and the control group (the other subjects) based on CRR of WSS, WSF and TUGSS, and DTE of WSS, respectively. Grouped by CRR of WSS, there were statistical differences in years of education (*p* = 0.003) and Fazekas scores of DWMH (*p* = 0.046) between the impairment group and the control group. Grouped by CRR of WSF, significant differences were found in diabetes (*p* = 0.013), hyperlipidemia (*p* = 0.044), antidiabetic drug (*p* = 0.037), presence or absence of lacuna (*p* = 0.046), Fazekas scores of DWMH (*p* < 0.001) and PVH (*p* < 0.001) and severity of BG-PVS (*p* = 0.044) between the impairment group and the control group. As for DTE of WSS, statistical differences were found in education (*p* = 0.003) and DWMH (*p* = 0.046) between two groups. In each grouping method, statistical differences were found in scores of MMSE (*p* < 0.05) and MoCA (*p* < 0.05) between the impairment group and the control group.

Grouped by CRR of TUGSS, there were significant differences in education (*p* = 0.014), smoking (*p* = 0.022), hyperlipidemia (*p* = 0.011), lipid-lowering drug (*p* = 0.011), MMSE (*p* < 0.001) and MoCA (*p* < 0.001) scores between the impairment group and the control group (Table 3). Binary logistic regression analysis showed that the presence of CMB was an independent risk factor for the impairment group of CRR of TUGSS (*OR*, 2.54; 95% CI, 1.11–5.82; *p* = 0.027) with the adjustment of age, sex, education, smoking, hyperlipidemia, lipid-lowering drug, MMSE and MoCA (Table 4).

**Table 3:** Comparison of characteristics between the impairment group and the control group classified by CRR of TUGSS.

	**Impairment group (*n* = 58)**	**Control group (*n* = 153)**	***p***
Age^a^, year	69.50 (63.00, 76.25)	70.00 (65.00, 75.50)	0.784
Men, *n* (%)	29 (50.00)	87 (56.86)	0.371
Years of education^a^, year	9 (6, 12)	9 (9, 13)	0.014^*^
Height^a^, m	1.65 (1.58, 1.70)	1.65 (1.60, 1.70)	0.630
Weight^a^, kg	66.00 (60.00, 75.00)	69.00 (60.50, 76.50)	0.174
MMSE^a^	26 (24, 28)	28 (27, 29)	<0.001^*^
MoCA^a^	23 (20, 25)	25 (23, 26)	<0.001^*^
Smoking, *n* (%)	11 (18.97)	54 (35.29)	0.022^*^
Drinking, *n* (%)	7 (12.07)	29 (18.95)	0.235
Hypertension, *n* (%)	43 (74.14)	104 (67.97)	0.385
Diabetes mellitus, *n* (%)	15 (25.86)	48 (31.37)	0.435
CAD, *n* (%)	10 (17.24)	40 (26.14)	0.175
History of stroke/TIA, *n* (%)	11 (18.97)	35 (22.88)	0.539
Hyperlipemia, *n* (%)	13 (22.41)	63 (41.18)	0.011^*^
Blood pressure-lowering medication, *n* (%)	35 (60.34)	94 (61.44)	0.884
Antiplatelet/anticoagulant drug, *n* (%)	13 (22.41)	53 (34.64)	0.087
Antidiabetic drug, *n* (%)	13 (22.41)	47 (30.72)	0.232
Lipid-lowering drug, *n* (%)	11 (18.97)	57 (37.25)	0.011^*^
CSVD MRI markers			
Presence of lacune, *n* (%)	25 (43.10)	58 (37.91)	0.490
Presence of CMB, *n* (%)	26 (44.83)	42 (27.45)	0.016^*^
PVH^b^	1 (1–3)	1 (1–3)	0.812
DWMH^b^	1 (0–3)	1 (0–3)	0.198
BG-PVS^b^	1 (1–4)	1 (1–4)	0.332
CSO-PVS^b^	2 (1–4)	2 (1–4)	0.302
PA ^b^	1 (0–2)	1 (0–2)	0.903

*CRR, correct response rate; TUGSS, timed up and go and serial subtraction; MMSE, mini-mental state examination; MoCA, Montreal cognitive assessment; CAD, coronary artery disease; TIA, transient ischemic attack; CSVD, cerebral small vessel disease; MRI, magnetic resonance imaging; CMB, cerebral microbleed; PVH, periventricular hyperintensity; DWMH, deep white matter hyperintensity; BG, basal ganglia; CSO, centrum semiovale; PVS, perivascular space; PA, posterior atrophy*.

a *Median (quartiles)*.

b *Median (range)*.

* *P < 0.05*.

**Table 4:** Binary logistic regression analyses of the relationship between the presence of CMB and the cognitive performance on dual tasks based on different grouping methods.

**Grouping method**	**CMB**	**Impairment group**	**Control group**	***p***	***OR* (95% CI)**	***p***
CRR of WSS	No CMB, *n*	31	112	0.020	1.79 (0.85, 3.74)	0.125^a^
	CMB, *n*	25	43			
CRR of WSF	No CMB, *n*	30	113	0.044	1.15 (0.51, 2.57)	0.742^b^
	CMB, *n*	23	45			
CRR of TUGSS	No CMB, *n*	32	111	0.016	2.54 (1.11, 5.82)	0.027^*^^c^
	CMB, *n*	26	42			
DTE of WSS	No CMB, *n*	31	112	0.020	1.79 (0.85, 3.74)	0.125^d^
	CMB, *n*	25	43			

*CMB, cerebral microbleed; OR, odds ratio; WSS, walking and serial subtraction; WSF, walking and semantic fluency; TUGSS, timed up and go and serial subtraction; CRR, correct response rate; DTE, dual-task effect*.

a *Adjusting for age, sex, education, deep white matter hyperintensity, MMSE and MoCA*;

b *adjusting for age, sex, education, diabetes, hyperlipidemia, antidiabetic drug, lacuna, deep white matter hyperintensity, paraventricular hyperintensity, basal ganglia perivascular space, MMSE and MoCA*;

c *adjusting for age, sex, education, smoking, hyperlipidemia, lipid-lowering drug, MMSE and MoCA*;

d *adjusting for age, sex, education, deep white matter hyperintensity, MMSE and MoCA*.

* *P < 0.05*.

**Figure 1:**
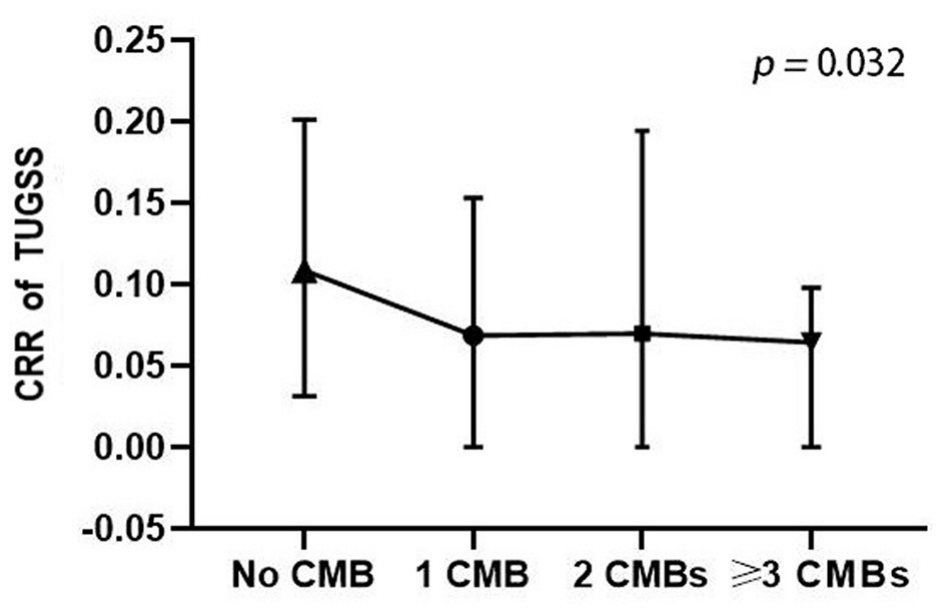
Comparison of CRR of TUGSS among four CMB number grades (Kruskal-Wallis H test). CRR, correct response rate; TUGSS, timed up and go and serial subtraction; CMB, cerebral microbleed.

Participants were further classified into four grades according to the number of CMB lesions. There were 143 participants without CMB, 32 participants with 1 CMB, 12 participants with 2 CMB lesions and 24 participants with more than 2 CMB lesions. A significant difference was found in CRR of TUGSS among four CMB number grades (*p* = 0.032) (Figure 1). Multiple linear regression analysis showed that CRR of TUGSS decreased with the increase of CMB grades (β, −0.144; 95% CI, −0.027, −0.002; *p* = 0.028) after the correction of age, sex, education, smoking, hyperlipidemia, lipid-lowering drug, MMSE and MoCA.

Moreover, binary logistic regression analysis suggested that the presence of CMB was not an independent risk factor for the impairment group on CRR of WSS (*OR*, 1.79; 95% CI, 0.85–3.74; *p* = 0.125), CRR of WSF (*OR*, 1.15; 95% CI, 0.51–2.57; *p* = 0.742) or DTE of WSS (*OR*, 1.79; 95% CI, 0.85–3.74; *p* = 0.125) after adjusting confounding factors respectively (Table 4).

## Discussion

When people perform a cognitive task and a motor task at the same time, there will be competition or interference between different tasks leading to the deterioration in one or both task performances. This is known as cognitive-motor interference, a specific kind of DT interference (Leone et al., 2017). The underlying mechanisms are still unclear. Several theories exist to explain it in humans (Pashler, 1994; Leone et al., 2017): (1) the capacity-sharing theory, which means limited resources must be reallocated between two tasks when people are performing them at the same time; (2) the bottleneck theory, which means when two tasks need the same mechanism, a bottleneck occurs, and one or both tasks will be delayed or impaired; and (3) the cross-talk theory, which refers to the content-dependent degradation or outcome conflict of two tasks, and theorists have usually favored that it is more difficult to perform two tasks even if they involve similar information.

Based on these theories, DT tests have been widely used to study the cognitive-motor interference and the risk of falls in different populations, such as patients with stroke, Alzheimer's disease, Parkinson's disease, or multiple sclerosis (Mofateh et al., 2017; Feld et al., 2018; de Oliveira Silva et al., 2020; Penko et al., 2020). Some studies found that old people had greater DT costs compared to young adults, and this deficit was related to the high incidence of falls in the elderly (Lindenberger et al., 2000; Bock, 2008; Pothier et al., 2015; Papegaaij et al., 2017; Uematsu et al., 2018). An age-related decrease in gait and balance function was also found in studies, which was in greater need of “attentional resources” (Lindenberger et al., 2000; Pothier et al., 2015; Papegaaij et al., 2017). In recent years, more and more studies have focused on the role of CSVD in the decline of DT performance in old people. A recent cross-sectional study found that WMH volume was related to slower gait speed and reduced stride length under DT conditions in dementia patients (Hairu et al., 2021). A study based on community-dwelling older people suggested the negative correlation between deep WMH volumes and the walking speed on DT conditions, which was mediated in part by global cognition and executive abilities specifically (Ghanavati et al., 2018). However, another study showed the opposite result that deep WMH was associated with impaired gait velocity of the single TUG test rather than DT walking (Hashimoto et al., 2014). Most studies have focused on gait disorders in the DT, and no studies have specifically targeted the DT performance of CMB patients. One study has collected the data of CMB, but the number of CMB patients was too small to find a difference between the gait disturbance group and the normal group (Hashimoto et al., 2014).

In this study, the combined protocol of various DT tests and single tasks was used to describe the feature of cognitive performances on DT of CMB patients. The results showed that the global cognitive function of CMB patients was worse than that of subjects without CMB, which was consistent with previous studies (Akoudad et al., 2016; Li et al., 2021a). Compared to subjects without CMB, CMB patients had worse cognitive performances on the DT condition in CRR of WSS, WSF and TUGSS, and DTE of WSS. In CMB patients, cognitive performances in two subtraction-DT tests were worse than those in single subtraction tasks, although the subjects were asked to perform two tasks without prioritization. Logistic regression analysis showed that the presence of CMB was a risk factor for the impairment group on CRR of TUGSS, independent of education, basic cognition and other confounders. These results also further confirmed the significant influence of CMB on cognitive function of the elderly.

As for the design of DT paradigm, the difficulty of DT can be changed by adjusting the level of complexity and novelty of each task (McIsaac et al., 2015). Verbal fluency and serial subtraction are widely used in CSVD patients to examine the sustained attention, information processing speed and executive function. One prior study has reported that the two tests are recommended for the assessment of TUG-cognitive task in stroke patients (Pumpho et al., 2020). Besides, two motor tasks of different levels of difficulty were used in this study, including 8-m walking and TUG tests. TUG is reliable and sensitive to detecting cognitive and mobility disorders, and can provide information about the gross motor function which is important for the maintenance of mobility in everyday tasks (Montero-Odasso et al., 2019). According to our results, CMB was an independent risk factor for the impairment group on CRR of TUGSS, where the performance worsened with the increasing degree of CMB burden. Thus, we propose that TUGSS may be a suitable DT test for evaluating the ability of CMB patients.

The underlying pathophysiological mechanisms of CMB-related cognitive and motor impairment have not been fully elucidated. The direct damage of CMB lesions on focal brain tissues may cause myelin loss, neuronal loss and variable extent of gliosis, and lead to the brain function disorder. The remote effects manifested by white matter microstructure changes and cortical thinning may also be an indirect damage to brain function caused by CSVD (Ter Telgte et al., 2018). Besides, CMBs are associated with the impairment of brain network, including longer path length and less global efficiency than people without CMB (Heringa et al., 2014; Reijmer et al., 2015). Another study in patients with cerebral amyloid angiopathy suggested that the brain network impairment worsened from posterior to frontal connections (in the fractional anisotropy) with the increasing disease severity (Reijmer et al., 2016). Moreover, a synergistic effect was found between CSVD and other neurodegenerative pathologies (such as Alzheimer's disease and Parkinson's disease) on patients' dysfunctions (Ter Telgte et al., 2018). Consequently, CMB lesions may lead to cognitive or motor disorders through the direct or indirect damage to the brain parenchyma and brain networks. Many prospective studies on the single cognitive task have demonstrated that the CMB is a crucial risk factor for cognitive deterioration and dementia (Akoudad et al., 2016; Ding et al., 2017). Our results expanded the above conclusion that CMB patients also had worse cognitive and gait performances on DT conditions than those without CMBs, especially in the decreased CRR and shortened stride length in TUGSS test. According to the therapy of reserve mechanisms (Ter Telgte et al., 2018), we speculated that for CMB patients with impaired reserve ability, the additional motor task on the basis of the single cognitive task would cause an aggravation of the cognitive dysfunction. On the other hand, the damaged brain tissue caused by CMB lesions made the cognitive-motor interference more significant. It might be related to the limited resources reallocated between two tasks and an inability to process the attentional demand of each task accurately (Leone et al., 2017; Ma et al., 2021). However, whether the influence of CMBs on motor function under DT conditions is directly caused by the lesions or mediated by cognitive dysfunction is an interesting question that needs further consideration and verification.

To our knowledge, this is the first study targeting the influence of CMB lesions on the cognitive performance under DT conditions in old adults. And the compound protocol of global cognition assessments, basic gait tests and different DT designs makes the functional assessment more diversified in this study. Moreover, the IDEEA, a microcomputer-based portable gait analysis system and physical activity monitor, has advantages in the detection and analysis of multiple gaits, postures, limb movements, and the energy expenditure with great accuracy (Huddleston et al., 2006).

However, there are still some limitations to this study. This is a single-center cross-sectional study, and the sample size is relatively small. So, we did not perform the subgroup analysis based on the CMB location or cognitive domains. Not all the gait parameters were included in the analysis, because this study paid more attention to the cognitive performance on DT that other studies had focused less on in CSVD patients. In the future, the sample size will be further expanded to conduct the interested subgroup analysis, and the motor function such as gait and balance will be complementally analyzed. In addition, those semi-quantitative scales were relatively subjective for the assessment of CSVD imaging markers. It may have some impact on the effectiveness of proving the tendency that cognitive performances on DT are changing with the severity of CMB burden. Further studies with advanced imaging techniques and post-processing methods may be of great value to provide more information and solve this problem.

## Conclusion

The presence of CMB was an independent risk factor for the cognitive impairment group of TUGSS on the DT condition, where performance deficits increased in proportion to the degree of CMB burden. Failing to consider the effect of DT may lead to an underestimation of the difficulties in CMB patients' daily life, including those with mild impairments. In the future, more large-scale studies with longitudinal designs are needed to clarify the causality between CMB and DT performances and to explore the mechanisms using multimodal imaging techniques and experimental sciences.

## Data Availability Statement

The raw data supporting the conclusions of this article will be made available by the authors, without undue reservation.

## Ethics Statement

The studies involving human participants were reviewed and approved by the Ethics Committee of Beijing Chao-yang Hospital, Capital Medical University. The patients/participants provided their written informed consent to participate in this study.

## Author Contributions

XL and WH contributed to conception and design of the study. XL, SY, WQ, YH, and QH collected the data. XL, YL, and SY performed the statistical analysis. XL wrote the first draft of the manuscript. WH, WQ, and LY contributed to the critical revision. All the authors contributed to manuscript revision, read, and approved the submitted version.

## Conflict of Interest

The authors declare that the research was conducted in the absence of any commercial or financial relationships that could be construed as a potential conflict of interest.

## Publisher's Note

All claims expressed in this article are solely those of the authors and do not necessarily represent those of their affiliated organizations, or those of the publisher, the editors and the reviewers. Any product that may be evaluated in this article, or claim that may be made by its manufacturer, is not guaranteed or endorsed by the publisher.
